# Genetic diversity and structure of the narrow endemic *Seseli farrenyi* (Apiaceae): implications for translocation

**DOI:** 10.7717/peerj.10521

**Published:** 2021-02-04

**Authors:** Núria Garcia-Jacas, Jèssica Requena, Sergi Massó, Roser Vilatersana, Cèsar Blanché, Jordi López-Pujol

**Affiliations:** 1 Botanic Institute of Barcelona (IBB, CSIC-Ajuntament de Barcelona), Barcelona, Catalonia, Spain; 2 Departament de Biologia Animal, Biologia Vegetal i Ecologia, Facultat de Biociències, Universitat Autònoma de Barcelona, Bellaterra, Catalonia, Spain; 3 Laboratori de Botànica, Facultat de Farmàcia i Ciències de l’Alimentació, Universitat de Barcelona, Barcelona, Catalonia, Spain

**Keywords:** Conservation, Extremely narrow endemic, Genetic diversity, Iberian Peninsula, *Seseli farrenyi*, Microsatellites, Recovery plan, Threatened, Reinforcement, Fragmentation

## Abstract

*Seseli farrenyi* (Apiaceae) is an extremely narrow endemic plant, which is considered as one of the species of most conservation concern in Catalonia (NW Mediterranean Basin). Given the accelerated fragmentation and reduction of population size (of over 90%), the environmental agency of Catalonia is currently preparing a recovery plan that includes reinforcements of the extant populations. The present study is aimed at providing the necessary knowledge to carry out genetically-informed translocations, by using microsatellites as genetic markers. Fourteen microsatellites have been specifically developed for *S. farrenyi*, of which nine have been used. Besides the extant natural populations, the three ex situ collections that are known to exist of this species have also been studied, as they would be the donor sources for translocation activities. Our main finding is that levels of genetic diversity in the natural populations of *S. farrenyi* are still high (*H*_e_ = 0.605), most likely as a result of a predominantly outcrossing mating system in combination with the limited time elapsed since the population decline. However, population fragmentation is showing the first genetic signs, as the values of genetic differentiation are relatively high, and two well-differentiated genetic lineages have been found even in such a narrow geographic range. These genetic results provide important information when designing conservation management measures.

## Introduction

Surveying genetic data through neutral markers provides very detailed information that may have important conservation implications in plant species, including effective population size (*N*_e_), mating systems, gene flow, clonal structure, colonization history, or the occurrence of evolutionarily significant units, among others. Therefore, genetic patterns should be central in management and recovery plans (Laikre, 2010; Ralls et al., 2018; Laikre et al., 2020). Detailed knowledge of the levels and genetic structure of plant populations/species is key to the objectives of representativeness and maximization of conservation resources (Ottewell et al., 2016; Chung et al., 2020). Leaving aside demographic considerations (Lande, 1988), in the absence of genetic data more populations would require protection or sampling for ex situ conservation, as a precaution, in order to preserve sufficient levels of genetic variability (Neel & Cummings, 2003). This implies higher economic costs and more complex logistics.

Ignoring genetic factors can lead, moreover, to inadequate species management (Frankham, 2005; Bubac et al., 2019). For example, translocations for which genetic diversity of the target species was known have been much more successful than those for which this previous knowledge did not exist (reviewed in Godefroid et al. (2011)). The lack of data on species’ genetic variation has often condemned the actions of conservation and restoration to failure. The materials used for reintroduction may be inappropriate if they come from populations that are very different from a genetic point of view, as this can lead to the breakdown of the co-adapted gene complexes and, subsequently, to outbreeding depression (Fenster & Dudash, 1994; Frankham et al., 2011). Another risk of ignoring the genetic structure in the conservation plans is to overlook the existence of populations genetically unique. These populations are relevant from an evolutionary point of view because they might constitute either evolutionarily significant units (ESU; Moritz, 1994) or relevant genetic units for conservation (RGUC; Caujapé-Castells & Pedrola-Monfort, 2004).

**Figure 1:**
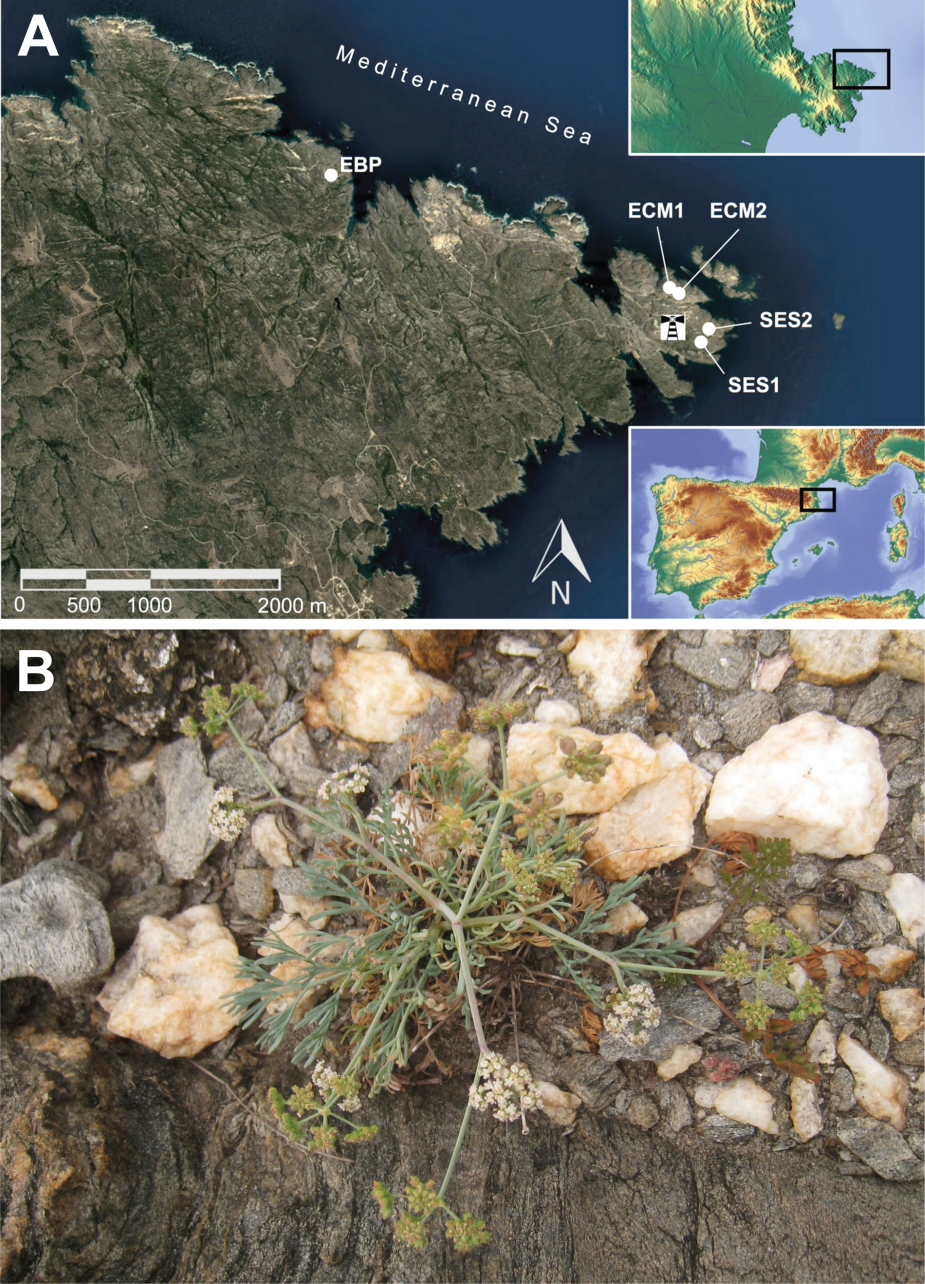
Geographic location of the extant populations of *Seseli farrenyi* (A), and flowering individual of *S. farrenyi* from EBP population (B). As this species is very threatened and thus particularly sensitive to collecting, the geographic location of populations in (A) is only approximate. The orthophotomap is from Institut Cartogràfic i Geològic de Catalunya, under license CC BY 4.0, the inset maps are from © Collaborators from OpenStreetMap. Photograph of (B) Jordi López-Pujol.

*Seseli farrenyi* Molero & J. Pujadas (Apiaceae) is an endemic species with a very small distribution range, being considered by the local environmental authorities as one of the species of most conservation concern in Catalonia (NW Mediterranean Basin). This small Apiaceae was discovered in the late 1970s (Molero & Pujadas, 1979) in the easternmost point of the Iberian Peninsula, Cape Creus (Fig. 1A). During the 1990s two new geographically close locations were reported (Franquesa, 1995), and the total population size was estimated to be around 2,000 individuals in 1999 (López-Pujol, 2000). Despite the surprisingly high levels of genetic diversity detected with allozymes in samples collected in the autumn of 2000 (*H*_e_ = 0.297; López-Pujol et al., 2002), the species was cataloged as “Endangered” (EN) according to the IUCN (2001) criteria both in the Spanish (Rovira et al., 2003) and in the Catalan (Sáez, Aymerich & Blanché, 2010) red books, mainly on the basis of its extremely small range (both in terms of extent of occurrence and area of occupancy) and population size. A new census in 2010 (López-Pujol et al., 2010) revealed a decline of over 90% of the total number of individuals to less than 150 due to unknown reasons; thus, the species can be regarded as an “extremely narrow endemic” (ENE; López-Pujol et al., 2013) plant. In accordance with this dramatic shrinkage, the species was re-evaluated against all the criteria, transferring it to the category of highest threat, “Critically Endangered” (CR). The environmental agency of Catalonia immediately launched a demographic monitoring (but with no accompanying conservation measures) that seems to reveal a stabilizing trend and even a certain demographic recovery (Table 1) for no apparent reason, again. However, the species is still well below the minimum viable population size (MVP; Allendorf & Luikart, 2007), and there is again some declining since 2018. Both the area of occupancy and extent of occurrence remain extremely low for the species (0.0023 and 0.0384 km^2^, respectively; CEBCAT-La Balca, 2016).

**Table 1:** Censuses performed since 1979 in all known *Seseli farrenyi* populations.

Population	1979^1^	1999^2^	2004^3^	2007^4^	2008	2010^7^	2011^8^	2012^9^	2013^9^	2015^10^	2016^11^	2017^11^	2018^11^	2019^12^
SES1	>500	90	0	0	0^5^	0	0	0	4	9	9	9	11	1
SES2	nd	nd	nd	nd	nd	4	4	4	12					8
ECM	nd	716	nd	nd	>40^6^	21	42	61	46	92	81	49	61	59
EBP	nd	1,260	nd	nd	nd	123	166	103	146	166	215	235	212	169
Total	na	2,066	na	na	na	148	212	168	208	267	305	293	284	237

**Notes:**

1 Molero & Pujadas (1979).

2 López-Pujol (2000).

3 J. Font (2005, personal communication).

4 J. López-Pujol (2007, personal observations).

5 C. Blanché, J. López-Pujol & M. Bosch (2008, personal observations).

6 Inferred from Martinell (2011).

7 López-Pujol et al. (2010).

8 Martinell (2011).

9 Martinell & López-Pujol (2014).

10 CEBCAT-La Balca (2016). In this study, there are no census data separately for each of the two SES subpopulations.

11 Saura-Mas (2018). In this study, there are no census data separately for each of the two SES subpopulations.

12 S. Saura-Mas & G. Carrión (2019, personal communication).

na, not applicable; nd, no data.

To reverse this trend and to avoid the extinction of *S. farrenyi*, a recovery plan should be urgently implemented. Ideally, this plan would include translocation activities, mainly reinforcements of the extant populations given their declining trend. Such a plan, which is mandatory (as the species is considered legally protected with the highest status, both by Catalonian and Spanish law; Departament de Medi Ambient i Habitatge, Generalitat de Catalunya, 2008; Ministerio de Agricultura, Alimentación y Medio Ambiente, 2015), is currently being drafted by the environmental agency of Catalonia, and might include reinforcements with plant material (seeds and/or seedlings) coming from the extant ex situ collections. The present study is, thus, aimed to provide the necessary knowledge to carry out genetically-informed translocations and to increase the chance of success in these activities (Godefroid et al., 2011). We are employing nuclear microsatellites or simple sequence repeats (nSSR) which, together with allozymes, have the advantages of codominant inheritance and reproducibility; however, SSR are much more variable than allozymes, which makes them to be probably the most suitable marker for conservation genetics, as they are capable of detecting subtle genetic differences (Finger & Klank, 2010; Abdul-Muneer, 2014). As no microsatellites for *S. farrenyi* (or for any other species of *Seseli*) have been published to date, developing a set of nSSR has also become a primary aim of the present study.

Our specific aims are the following: (1) developing specific microsatellite markers for the endangered *Seseli farrenyi*; (2) surveying the levels and distribution of the genetic diversity within and among its extant wild populations, as well as of the three ex situ populations; (3) comparing the nSSR genetic variation with the results of López-Pujol et al. (2002) using allozymes; and (4) providing genetically-oriented suggestions on how translocations should be carried out to maximize their success.

## Materials and Methods

### Study species

*Seseli farrenyi* is a small (up to 30 cm) monocarpic perennial herb with a basal rosette of pinnatisect leaves, and produces abundant compound umbel rays with small white flowers and ovoid seeds of 2.2–3 × 2–2.5 mm (Molero & Pujadas, 1979; Fig. 1B). It is a protandrous and mostly outcrossing plant (albeit self-compatible), visited by a great variety of insects (wasps, small bees, ants, flies, syrphid flies, beetles and stink bugs; Rovira et al., 2002). It reproduces exclusively by sexual means. Based on data from natural populations (observed in late 1990s–early 2000s), only a small number of individuals bloom every year (around 27%; Rovira et al., 2002); this percentage seems to have dropped below 20% according to the demographic monitoring carried out since 2010 (Martinell & López-Pujol, 2014; CEBCAT-La Balca, 2016), with some years with a particular paucity of reproductive individuals (3% in 2014 and 3.4% in 2019; S. Saura-Mas & G. Carrión, 2019, personal communication). Individuals are short-lived, mostly blooming between 3 and 5 years according to both field observations and greenhouse experiments (Martinell & López-Pujol, 2014). Seeds of *S. farrenyi* do not have morphological adaptations for their dispersal by anemochory; field data indicate that most seeds fall near the mother plant (Martinell & López-Pujol, 2014). There is no soil seed bank and, in addition, seed germination and seedling recruitment seem to be highly episodic (Martinell & López-Pujol, 2014). *Seseli farrenyi* is a diploid species with 2*n* = 18 (Fernández-Casas, Molero & Pujadas, 1979). The species grows in fissures of schistous rocks in coastal cliffs, on weakly acidic and sandy soils. The plant community to which *S. farrenyi* belongs, *Armerietum ruscinonensis* Br.-Bl., Roussine et Nègre 1952, also includes other endemic species such as *Armeria ruscinonensis* Girard and *Limonium tremolsii* (Rouy) P. Fourn. According to the EU Habitat Directive, it corresponds to Habitat 18.221+ (CORINE) and to HCI (Habitat of Community Interest) no. 1240, which should be object of conservation measures (European Comission, 2013).

### Plant material

Given the low population size of *S. farrenyi*, a special effort was made in sampling: all individuals with enough size to assure survival were sampled, and collected leaf tissue was kept to the minimum. All the locations were sampled (in summer 2017): 38 individuals from the largest population, EBP; 21 individuals from ECM (15 and six individuals from subpopulations ECM1 and ECM2, respectively), and three individuals from SES2 (Fig. 1A; Table 2). As the individuals tend to be spatially aggregated within each population/subpopulation (Martinell & López-Pujol, 2014), we made sure to collect at least one individual from all patches. The *locus classicus* (SES1) near the lighthouse (Fig. 1A) could not be sampled because the individuals were too small. However, we have included in the present study a dried specimen (from the personal herbarium of J.-P. Reduron, accession no. 97215) sampled in 1999 from the living collection of *S. farrenyi* of the Botanical Garden of Mulhouse (France), coming from seeds collected by R. Auriault at the *locus classicus* in 1983. The population (SES1) would have been much larger at that time, as its size was estimated at some 500 individuals in late 1970s (Molero & Pujadas, 1979; Table 1). We have also included individuals from the two known ex situ collections of *S. farrenyi*: 25 individuals from the living collection from the Botanical Garden of Barcelona (coded as JBB) and 12 from the plants grown in the greenhouses of the Sigma-Olot Consortium (coded as OLO; Table 2). Both ex situ populations have their origin in a seed collection campaign carried out in 2008 by the Marimurtra Botanical Garden of Blanes (as part of a seed collection program of the endangered flora of Catalonia), which took place in ECM, but in a patch that today does not exist (it was located between the current ECM1 and ECM2). It should be noted that these 37 sampled individuals are grown from the original seeds collected in 2008 (i.e., they represent the F1 generation). Thus, a total of 100 individuals have been collected, and they were kept in paper bags with silica gel until DNA extraction. Permissions to access the populations and sample the plant material were provided by the Catalonian Government (*Generalitat de Catalunya*) under the contract no. PTOP-2017-827. Given the threatened and protected status of this species, no vouchers have been collected this time; however, individuals coming from all sampled populations of this species were extensively vouchered in the past, with good specimens deposited in a high number of herbaria (including BC, BCN, COFC, FCO, LEB, MA, MAF, MGC, SALA, or SEV).

**Table 2:** Main parameters of genetic diversity for the natural populations (in situ) and ex situ of *Seseli farrenyi*.

Population	Sample size	MLG	*P*_95_ (%)	*A*	AR	TA/PA/RA^3^	FAN^4^	*H*_o_/c *H*_o_	*H*_e_/c *H*_e_	*F*_IS_/c *F*_IS_	DIC (*nfb*)	DIC (*nb*)
In situ populations												
SES2	3	3	77.8	2.11	1.42	19/0/na	0.035/0.031	0.370/0.412	0.422/0.382	0.149/0.077	**64.139**	64.245
ECM1	15	15	88.9	4.56	1.60	41/0/5	0.174/0.175	0.342/0.677	0.603/0.647	0.441*/0.000	566.578	**564.383**
ECM2	6	6	88.9	3.89	1.64	35/1/na	0.163/0.049	0.315/0.387	0.638/0.606	0.530*/0.355*	**235.619**	240.475
ECM^1^	21	21	88.9	5.44	1.64	49/2/12	0.184/0.189	0.335/0.703	0.639/0.676	0.482*/0.000	864.953	**862.537**
EBP	38	38	100	7.89	1.79	71/26/21	0.196/0.036	0.448/0.499	0.787/0.785	0.434*/0.381*	**1,963.959**	1971.013
Mean^2^		15.5	**88.9**	**4.61**	**1.61**	**41.5/6.75/13**	**0.142/0.073**	**0.369/0.494**	**0.613/0.605**	**0.389/0.203**		
Ex situ populations												
SES1	1	1	44.4	1.44	1.44	13/0/na	0.001/na	0.444/na	0.444/na	0.000/na	na	na
JBB	25	25	88.9	5.78	1.63	52/6/19	0.196/0.120	0.302/0.517	0.626/0.658	0.522*/0.232*	**979.643**	985.007
OLO	12	12	88.9	3.89	1.58	35/0/2	0.151/0.087	0.316/0.464	0.579/0.588	0.466*/0.230*	**409.100**	416.589
Mean		**12.7**	**74.07**	**3.70**	**1.55**	**33.3/2/10.5**	**0.116/0.104**	**0.354/0.491**	**0.550/0.623**	**0.329/0.231**		

**Notes:**

1 ECM is the combination of ECM1 and ECM2.

2 ECM1 and ECM2 are treated as populations (thus, ECM is not considered in the calculation of means).

3 The occurrence of rare alleles is only possible for those populations with a minimum sample size of 11 individuals.

4 On the left, using FreeNa; on the right, using INEst.

* *P* < 0.05.

MLG, total number of multi-locus genotypes; *P*_95_, percentage of polymorphic loci (95% criterion); *A*, mean number of alleles per locus; AR, allelic richness (adjusted to *N* = 1); TA; total number of alleles per population; PA, number of private alleles per population; RA, number of rare alleles per population; FAN, frequency of null alleles; *H*_o_, observed heterozygosity; *H*_e_, unbiased expected heterozygosity; *F*_IS_, inbreeding coefficient. For *H*_o_, *H*_e_ and *F*_IS_, the “c” letter denotes that they are corrected values due to the presence of null alleles; DIC, deviance information criterion (in bold, the model which is more approximate to the reality); *nfb*, full model; *nb*, model without the inbreeding parameter; na, not applicable.

### DNA extraction and microsatellite development

Total genomic DNA was extracted using E.Z.N.A.^®^ SP Plant DNA Kit (Omega Bio-tek Inc., Norcross, GA, USA), following the manufacturer’s instructions. Microsatellite isolation was carried out by Genetic Marker Services (Brighton, United Kingdom; www.geneticmarkerservices.com). The protocol for enrichment of genomic library followed Letelier et al. (2015). For *S. farrenyi*, fifty-eight motif-positive clones were detected and sequenced, of which 22 contained useful repeat motifs sufficiently long as to design pairs of primers. The primer pairs were developed with amplification products that varied between 150 and 280 base pairs (bp) using the software Primer3 (Rozen & Skaletsky, 2000). To verify the effectiveness of the amplification of the primer pairs, the Polymerase Chain Reaction (PCR) was performed following the conditions and the profile in Letelier et al. (2015).

In all PCR products, specificity, active polymorphism, and null alleles were verified by high resolution agarose gel. Fourteen of the 22 loci showed clear and specific bands that were variable among seven individuals sampled from several populations (SES1, SES2, ECM1, ECM2, EBP and JBB); these 14 loci were selected and universal M13 tails were added for fluorescence labeling (Table 3).

**Table 3:** Characteristics of 14 microsatellite primers developed for *Seseli farrenyi*.

Locus	Primer sequence (5′→3′)	*T*_*a*_ (°C)	Repeat motif	Size range (bp)	*N*	GenBank accession no.
SFA2^1^	F: *GCG GGA ATA AGG AGG AGA GT R: ATT CGG GCA CCT TAA ATC CT	59	(AG)13	154–178	100	MT628686
SFA9	F: *CAA AAC AGG GGG TTA TCA TTG R: TTT GGG GAT CTT GTC CAT CT	59	(GA)9	192	7	MT628687
SFA12^1^	F: TTT GAA TTG AAT AGG TTG CAA GAA R: *TCT CGA CAG CTC AAC CGT AA	60	(GT)13(GA)16	194–236	100	MT628688
SFA15^1^	F: *ACC TCG GTA TCA TCA ACC TG R: ATC AAG GCC TTT CGA ATA CC	58	(GT)17	179–209	100	MT628689
SFA18^1^	F: *AGT AGC AAG GTC CGC GTA AA R: GGA AGC TGA GCT ATG CTG ATG	60	**(**CT)10	149–169	100	MT628690
SFA24^1^	F: *CTT CGA CAC TGC ACC AAA GA R: GAC ATC TTA ACG GTC CAA GAG C	60	(CT)14(CA)26	246–274	100	MT628691
SFA31^1^	F: *AGA GCT TTT GGG TTT GCG TA R: AAG ACC CCT TTC CAG CTT GT	60	(CT)12	216–224	100	MT628692
SFA32^1^	F: CCA ACA AAT CAA TTA ATC ACA CC R: *TGG GTC GAA TAT TAT GGA TGG	59	(AG)32	185–211	100	MT628693
SFA35^1^	F: *CGC AAG CAC AGA GGG ATA AT R: TGG GGG TAA TTG GTG CTC TA	60	(GT)17	186–204	100	MT628694
SFA46	F: TCC CAT TTG TTA GAA CCT GTT G R: *ACA ATA CTT GGG TCT CTG TCG	56	(AG)23	228	7	MT628695
SFA47	F: *GCT ATG CGC CCG TAG TTT AC R: ACG CGT GGA CTA ACA TCT CTC	59	(GA)30	193	7	MT628696
SFA48^1^	F: *AAT AAA CTG GCG TGC AAT CA R: CAC TGG CAA TCC ATT ATG CTA A	59	(GT)10(GA)16	153–181	100	MT628697
SFA55	F: GTA CAT CAA GAT AAA TAT ACA CAA ACA R: *CGC CCT TGT AGT TTT CAT TA	55	(GA)28	212	7	MT628698
SFA57	F: *GCC CTT TCA ACA ATC ATC CA R: GTG AGT TCG TGG GTT GGT GT	60	(AC)14	149	7	MT628699

**Notes:**

1 Selected polymorphic loci.

* M13 tail (CACGACGTTGTAAAACGAC).

The 14 selected loci were tested with all the collected individuals, both from natural populations and cultivated ones (100 individuals in total). Amplifications were performed in 12 μL reaction containing 0.5 U AmpliTaq Gold polymerase (Applied Biosystems, Foster City, CA, USA), 2.0 mM MgCl_2_, 50 μM of each dNTP, 0.45 μM of the reverse primer, 0.012 μM of the extended forward primer, 0.45 μM of the labeled M13 primer (6-FAM, VIC, NED or PET; Applied Biosystems, Foster City, CA, USA) and 1 μL of total genomic DNA.

Finally, of the 14 loci selected, only nine loci were amplified for all the individuals sampled. The products of the PCR with fluorescence labeling of these nine primers were separated using an internal standard-size marker in an automatic sequencer ABI 3730xl DNA Analyzer (Applied Biosystems, Foster City, CA, USA) at the Madrid Science Park (Spain). Fragment analysis was performed using the GeneMarker v. 1.85 (SoftGenetics, LLC, State College, PA, USA).

### Genetic analyses

The individual genotypes of all the studied specimens were used for calculating allele frequencies by locus and population. The common statistics for population genetic studies were also estimated: total number of multi-locus genotypes (MLG); percentage of polymorphic loci when the most common allele had a frequency of <0.95 (*P*_95_); mean number of alleles per locus (*A*); allelic richness (AR, adjusted to the smallest sample size, *N* = 1 at SES1); number of total alleles per population (TA); number of private alleles (PA); number of rare alleles (those occurring at frequencies below 0.05) (RA); observed heterozygosity (*H*_o_); unbiased expected heterozygosity (*H*_e_) and Weir & Cockerham’s (1984) inbreeding coefficient (*F*_IS_). All these parameters were calculated using GenAlEx v. 6.1 (Peakall & Smouse, 2006) and Genetix v. 4.05 (Belkhir et al., 1996). Linkage disequilibrium between pairs of loci (at population level and across all populations) and deviations from Hardy–Weinberg equilibrium were assessed with the software GenePop v. 4.0.10 (Raymond & Rousset, 1995).

The programs FreeNA (Chapuis & Estoup, 2007) and INEst v. 2.2 (Chybicki & Burczyk, 2009) were used to make an exploratory estimation of the presence of null alleles at each locus. With FreeNA, the frequency of null alleles ranged from 0.000 for the locus 7 to 0.255 for the locus 8, with a mean of 0.131. The frequency of null alleles was slightly lower with INEst, ranging from 0.018 for the locus 7 to 0.191 for the locus 8, and averaging 0.083 (Table 4). As the frequency of null alleles in three out of nine loci was higher than 0.200 (below this value, bias introduced by null alleles is considered negligible; Dakin & Avise, 2004), INEst was used to get corrected values of *F*_IS_. Using a Bayesian approach, with 50,000 burn-in cycles and 500,000 Markov Chain Monte Carlo (MCMC) iterations in total, INEst was run for each population using two models. The first model (*nfb*) considered null alleles, inbreeding coefficients and genotyping failures. In the second model (*nb*) the inbreeding parameter was discarded. According to Chybicki (2017), the best model is the one with the lowest value of deviance information criterion (DIC, cf. Spiegelhalter et al., 2002); if the “full” model (*nfb*) fits the data better than the *nb* model, inbreeding should be considered as the “significant” component of the model and, thus, it explains the observed value of *F*_IS_. The INEst program was also used to calculate the parameters *H*_o_ and *H*_e_ corrected for the presence of null alleles, and to determine the statistical significance of the *F*_*IS*_ values for each locus and population (with 100,000 permutations).

**Table 4:** Null allele frequencies for each of the nine studied loci in *Seseli farrenyi*, using FreeNa and INEst programs for their estimation.

	SFA2 locus	SFA12 locus	SFA15 locus	SFA18 locus	SFA24 locus	SFA31 locus	SFA32 locus	SFA35 locus	SFA48 locus	Mean nine loci
Free NA	0.081	0.150	0.025	0.206	0.188	0.000	0.255	0.023	0.249	0.131
INEst*	0.061	0.076	0.022	0.123	0.102	0.018	0.191	0.022	0.132	0.083

**Note:**

* The SES1 population has not been considered in the estimation of null allele frequencies because the INEst software does not allow considering such small populations.

The occurrence of recent bottlenecks (in the last 2*N*_e_–4*N*_e_ generations, where *N*_e_ is the effective population size) in natural populations was explored with two different programs: (1) Bottleneck v. 1.2.02 (Piry, Luikart & Cornuet, 1999), by means of a sign test (Cornuet & Luikart, 1996) and a Wilcoxon signed-rank test (Luikart & Cornuet, 1998); and (2) INEst, by means of a *Z*-test and also a Wilcoxon signed-rank test. All tests were carried out under two different models, the infinite allele model (IAM) and the stepwise mutation model (SMM).

The program FreeNA was used to estimate Wright (1965) pairwise *F*_ST_ values, with and without the ENA (“excluding null alleles”) correction for null alleles (Chapuis & Estoup, 2007). The genetic structure of the species was evaluated using two approaches. First, the clustering software Structure v. 2.3.4 (Pritchard, Stephens & Donnelly, 2000), which uses a Bayesian algorithm, was employed, under the following conditions: 1 to 7 as the assayed number of clusters (*K*), 20 iterations per *K*, admixture model, correlated allele frequencies, no locprior, burn-in period of 100,000 and 1,000,000 MCMC replications. The most likely value of *K* was determined in two ways, as described in Garcia-Jacas et al. (2019): (1) the “plateau” method (Pritchard, Wen & Falush, 2010) and (2) by using the Δ*K* (Evanno, Regnaut & Goudet, 2005), as implemented in Structure Harvester (Earl & VonHoldt, 2012). The final image of this analysis was obtained using the programs Clumpp v. 1.1.2 (Jakobsson & Rosenberg, 2007) and Distruct v. 1.1 (Rosenberg, 2004).

Second, the software GenAlEx was used for performing a Principal Coordinate Analysis (PCoA) at the population level, based on codominant genotypic distances. To assess whether natural populations followed a pattern of isolation-by-distance, a Mantel test (1,000 permutations) between the matrix of pairwise genetic differentiation [*F*_ST_/(1 − *F*_ST_)] and the matrix of the log-transformed geographical distances was carried out with the software NTSYSpc v. 2.2 (Rohlf, 2009). Finally, the software BayesAss v. 1.3 (Wilson & Rannala, 2003) was used to estimate the recent (over 2–3 generations) migration rates (*m*) between natural populations, under the following conditions: 3,000,000 MCMC iterations with a burn-in of 999,999 iterations, sampling frequency of 2000, and delta set at 0.15 (the default value).

## Results

### Characterization of microsatellite markers for *Seseli farrenyi*

The 14 microsatellite markers characterized for *S. farrenyi* are the first developed for this species, and nine of them were used for population genetic studies reported in the present article. Properties of the 14 properly amplified polymorphic nSSR markers are summarized in Table 3. All microsatellites were dinucleotides, with the number of repeats motif ranging from nine to 32, and the GA-rich motifs the most recurrent in all repeat types. The PCR products were 149 to 274 bp in length. All primers had a theoretical melting temperature between 55 and 60 °C. The usual statistics for population genetic studies for the nine loci that amplified for all the individuals sampled are reported in the next sections.

### Levels and distribution of genetic diversity in *Seseli farrenyi*

The levels of genetic diversity within populations of *S. farrenyi* were considerable (*P*_95_ = 88.9%; *A* = 4.61; *H*_e_ = 0.605 for the in situ populations; Table 2). EBP was the most variable population (*H*_e_ = 0.785; Table 2). EBP was also the population that harbored more alleles (71 out of 92 detected alleles at the species level, taking into account both natural and ex situ populations), with a significant part of them (26 of 71 alleles, nearly 37%) being exclusive (Table 2; Table S1). The two ECM subpopulations, either analyzed individually or merged, showed intermediate levels of genetic diversity, and the ECM population as a whole harbored two exclusive alleles. The two ex situ populations JBB and OLO maintained similar levels of genetic diversity to ECM (the donor population), although the JBB population showed up to six exclusive alleles (Table 2; Table S1). Within the ex situ populations, two additional alleles appeared which had gone undetected in the natural populations: the allele 195 of SFA32 locus, shared between JBB and OLO, and the allele 210 of SFA12 locus, present in all ex situ populations.

Concerning *F*_IS_, all populations and subpopulations showed significant levels of inbreeding, in some cases with high values (e.g., ECM2 and JBB, with values over 0.500; Table 2). Only two subpopulations were not apparently inbred (SES1 and SES2), although this result was probably an artifact given the small sample sizes (*N* = 1 and *N* = 3, respectively; Table 2). Nevertheless, *F*_IS_ values largely fell when the distorting effect of the null alleles was suppressed, although some populations still showed an important excess of homozygotes (ECM2, EBP, JBB and OLO; Table 2); these four populations, as could be expected, were those which best fit the *nfb* model. The high levels of inbreeding in *S. farrenyi* were also exemplified by the values of *F*_IS_ at individual loci: 20 out of 54 possible cases showed a significant excess of homozygotes, whereas the remaining 34 were in agreement with Hardy–Weinberg expectations. Bottleneck test results suggested that all populations/subpopulations with the exception of the very small SES2 (*N* = 3) have suffered recent decreases in effective population size (Table 5).

**Table 5:** Results of the tests for identifying the occurrence of recent bottlenecks in the natural populations of *Seseli farrenyi*, using two different programs (Bottleneck and INEst).

Population	Bottleneck				INEst			
	Sign test		Wilcoxon test		*Z*-test		Wilcoxon test	
	IAM	SMM	IAM	SMM	IAM	SMM	IAM	SMM
SES2	0.535	0.511	0.656	0.766	na	na	na	na
ECM1	0.090	0.409	**0.004**	0.371	**0.013**	0.187	**0.004**	0.156
ECM2	0.250	0.443	**0.027**	0.578	**0.017**	0.125	**0.019**	0.073
ECM	**0.013**	0.406	**0.002**	0.629	**0.006**	0.277	**0.004**	0.273
EBP	0.064	0.544	**0.002**	0.500	**0.001**	0.448	**0.002**	0.151

**Note:**

IAM, infinite allele model; SMM, stepwise mutation model; na; not applicable. *P* < 0.05, in bold.

Several cases of genotypic linkage disequilibrium were found between pairs of loci across all populations (11 out of 36 possible cases). All loci except SFA2 were involved in the detected cases of linkage disequilibrium, and there were no specific pairs of loci overrepresented in this phenomenon. When analyzing population by population, all populations were affected except SES2 and ECM2 (though it has not been possible to apply the test to SES1 because of its insufficient population size), and the SFA2 locus was at least involved in two cases of linkage disequilibrium (one each for ECM and EBP),

The *F*_ST_ values were similar whether taking the null alleles into account or not (0.138 vs. 0.142 across loci considering all populations; 0.146 vs. 0.150 on average per locus; 0.181 vs. 0.151 on average by pair of populations). For this reason, the effect of null alleles on the patterns of genetic structure of *S. farrenyi* could be regarded as negligible. As expected, the matrix of pairwise *F*_ST_ (Table 6) showed values below 0.10 between the two ECM subpopulations and also between these and the two ex situ populations coming from the same ECM field location. In contrast, and unexpectedly, the highest value of differentiation was between SES1 and SES2, although this could be an artifact resulting from small sample sizes.

**Table 6:** Genetic differentiation between populations of *Seseli farrenyi* based on *F*_ST_, with and without ENA correction for null alleles (Chapuis & Estoup, 2007).

	SES2	ECM1	ECM2	EBP	SES1	JBB	OLO
*F*_ST_ using the ENA correction							
SES2	0.000						
ECM1	0.266*	0.000					
ECM2	0.260*	0.057ns	0.000				
EBP	0.278*	0.151*	0.121*	0.000			
SES1	0.426*	0.251*	0.187*	0.218*	0.000		
JBB	0.240*	0.032*	0.056*	0.152*	0.254*	0.000	
OLO	0.291*	0.072*	0.076*	0.168*	0.235*	0.009ns	0.000
*F*_ST_ without using the ENA correction							
SES2	0.000						
ECM1	0.255*	0.000					
ECM2	0.241*	0.069ns	0.000				
EBP	0.237*	0.174*	0.126*	0.000			
SES1	0.407*	0.177ns	0.065ns	0.083*	0.000		
JBB	0.195*	0.038ns	0.053*	0.166*	0.133ns	0.000	
OLO	0.273*	0.089*	0.067ns	0.174*	0.139ns	0.008ns	0.000

**Notes:**

ns, not significant

* *P* < 0.05.

**Figure 2:**
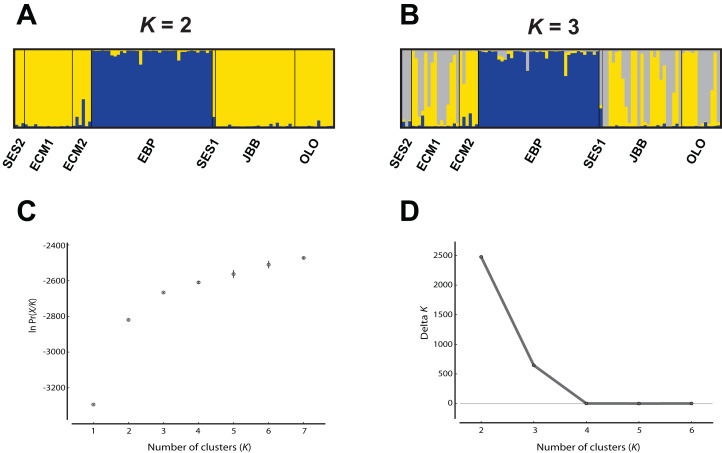
Analysis of the *Seseli farrenyi* populations using the Structure software. (A) Genetic structure assuming *K* = 2; (B) genetic structure assuming *K* = 3; (C) estimation of *K* with the “plateau” method (Pritchard, Wen & Falush, 2010); (D) estimation of *K* with the Δ*K* statistic of Evanno, Regnaut & Goudet (2005).

**Figure 3:**
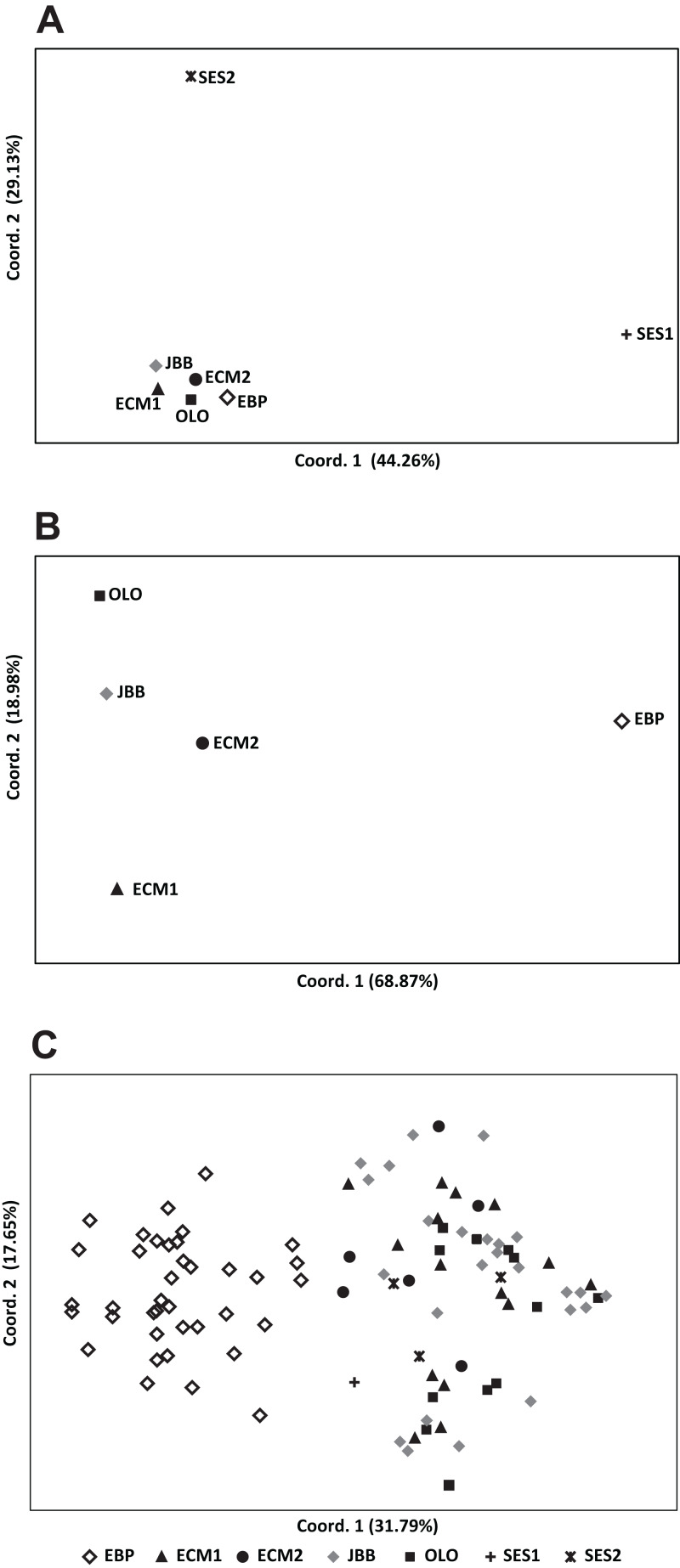
Principal coordinate analysis (PCoA) of *Seseli farrenyi* populations, performed with the genotypic distances obtained with GenAlEx. (A) PCoA with all the populations; (B) PCoA excluding the SES1 and SES2 subpopulations; (C) PCoA of all studied individuals of *Seseli farrenyi*.

While the Evanno, Regnaut & Goudet (2005) Δ*K* approach clearly showed *K* = 2 as the best genetic structuring scheme, the “plateau” method indicated that both *K* = 2 and *K* = 3 are plausible scenarios. In any case, two genetic lineages were configured: (1) the EBP population, which showed its own cluster both with *K* = 2 and *K* = 3, and (2) the rest of the populations, either assigned to the same cluster or showing admixture of two clusters (Fig. 2). The PCoA showed a pattern highly compatible with the Structure results, both at a populational and individual level; when the distorting effect of the smallest populations (SES1 and SES2) was discarded, the two genetic lineages depicted by Structure could be clearly differentiated within the two-dimensional space: (1) the western population (EBP) and (2) the eastern populations (the two ECM subpopulations plus the ex situ populations derived from these; Figs. 3A and 3B). At an individual level, the PCoA also discriminated the two genetic lineages, with the four individuals of SES population (the three individuals from SES2 and the descending individual from the harvested seeds of SES1 in 1983) appearing within the cloud of points of the eastern lineage (Fig. 3C).

The Mantel test found a low but not statistically significant correlation between genetic differentiation and the log of geographic distance (*r* = 0.335, *P* = 0.276), suggesting that there is no isolation-by-distance across all populations. Finally, the BayesAss analysis indicated that there were detectable levels of recent gene flow from ECM1 to SES2, and from ECM1 to ECM2 (Table S2).

## Discussion

### High genetic diversity in *Seseli farrenyi*

The levels of genetic diversity identified by microsatellites in the natural (in situ) populations of *S. farrenyi* should be regarded as high (*H*_e_ = 0.605) if they are compared with the reference values for plants in general (*H*_e_ = 0.420 and 0.620 for endemic species and for widespread ones, respectively; Nybom, 2004). The values of genetic variability observed here are also in agreement with a study using allozymes (conducted before the large population decline of the 2000s; López-Pujol et al., 2002), in which the authors reported exceptionally high levels of diversity (*H*_e_ = 0.297)—indeed among the highest values ever found for diploid plants using allozymes. The high levels of genetic diversity detected in *S. farrenyi* contradict the expectations for Extremely Narrow Endemic species (ENEs, defined in López-Pujol et al. (2013)). According to these authors, ENEs tend to show low genetic diversity (*H*_e_ = 0.057 with allozymes, *N* = 18), primarily due to bottlenecks related to the small population size. Reasons explaining the high genetic diversity in *S. farrenyi*, first revealed with allozymes and confirmed here with nSSR, might include: (1) an ancestor species with an exceptional genetic diversity; (2) a hybrid origin, with potential parents including the taxon for which *S. farrenyi* has been described as a subspecies—*S. elatum* L., widely distributed in the NE Iberian Peninsula, and perhaps one of the allopatric species for which *S. farrenyi* shows clear morphological and ecological affinities—*S. bocconei* Guss. and *S. praecox* (Gamisans) Gamisans; (3) the fact that *S. farrenyi* occurs within one of the main glacial Mediterranean refugia (SE Pyrenees; Médail & Diadema, 2009) and, within this refugium, it inhabits cliffs, a type of habitat that provides plants with high environmental stability and topographical diversity (Tzedakis et al., 2002; Médail & Diadema, 2009); and (4) having outcrossing as its main reproductive system (Rovira et al., 2002; see below).

The high levels of genetic variability of *S. farrenyi* are even more evident when observing the total number of MLG, as all the studied individuals except two from the ex situ populations (JBB9 and OLO7) show their own multi-locus genotype (Table 2); in other words, we have detected 99 MLG among the 100 sampled individuals. This is a very significant result, given that there is evidence of recent genetic bottlenecks within almost all populations (Table 5). Although some results should be treated with extreme caution (SES2 and ECM2 are below *N* = 10, which is the recommended minimum size to run the bottleneck tests), these genetic bottlenecks are in agreement with the demographic bottlenecks that have been observed for the last two decades, with reductions in the population size of up to 90% (Table 1). The most extreme case is the *locus classicus* (SES1 subpopulation), which would have been continuously declining since its discovery, 40 years ago. Moreover, some individuals would have existed in-between SES and ECM populations (J. Molero, 2002, personal communication), probably connecting them. It seems, therefore, that *S. farrenyi* has not suffered large losses in its genetic diversity in spite of the documented population size declining, at least since the early 2000s when the species was assessed with allozymes.

The dominance of outcrossing in the natural populations (Rovira et al., 2002), coupled with the short time elapsed after bottlenecks, would have prevented higher losses of genetic variability in *S. farrenyi*. First, outcrossing may “protect” somewhat against the effects of genetic drift because alleles are less likely to be eliminated; the reason is that in outcrossing species alleles tend to be shared in several individuals/populations thanks to increased gene (pollen) flow compared to selfing species (Hamrick & Godt, 1996). Second, when population reduction has taken place recently, large changes in the levels of genetic diversity are not expected because there have not been sufficient generations for the initial diversity to be substantially eroded (Zartman, McDaniel & Shaw, 2006; Chung et al., 2014, 2018). However, although *S. farrenyi* maintains a certain degree of “genetic health”, the current size of the populations cannot ensure its survival, even in the short term (see Blanché (2013) for more details), as they are well below the MVP. Extant populations of *S. farrenyi* range from *N* = 9 in SES to *N* = 169 in EBP (Table 1), not meeting, thus, the “500/5000 rule”, which is the result of applying the “50/500 rule” to the actual census size (*N*) instead to *N*_e_ (Traill et al., 2010).

### Levels of genetic diversity and inbreeding within populations

The more genetically variable population of *S. farrenyi* is EPB, both in terms of expected heterozygosity, total number of alleles, and private alleles. This is an expected result because EBP is the largest population in the last census (169 individuals, that is, 71% of the total known individuals of the species in its natural populations; Table 1). However, it should be taken into account that half of these exclusive alleles are rare (i.e., present at low frequencies, below 0.05) and, thus, at high risk of being lost. In consequence, sampling this population with the purpose of collecting seeds is of high priority, and such sampling should be mainly focused on the individuals that contain these exclusive alleles (or on the patches where these individuals occur). Private alleles may have important conservation implications because they might indicate local adaptation (Huenneke, 1991; Sjöstrand, Sjödin & Jakobsson, 2014), for example, alleles of neutral loci could be in linkage disequilibrium with alleles of adaptive loci; certainly, populations with private alleles are regarded as of high priority for conservation purposes (Petit, El Mousadik & Pons, 1998).

The ECM population as a whole harbors two exclusive alleles that, therefore, would not be captured in the seed collection carried out in this population in 2008 (the sampled individuals of both JBB and OLO ex situ populations were grown from seeds coming from this collection). As one might anticipate, the JBB and OLO populations show levels of genetic variation close to the donor population, although, unexpectedly, the JBB and OLO populations combined harbor seven exclusive alleles. It is likely that these seven alleles would have been extinguished since 2008, but it is also possible that they were not sampled for the present genetic study; given the threatened status of this species, the smallest individuals of each population were not sampled in the summer of 2017 to ensure their survival.

Most populations and subpopulations of *S. farrenyi*, including the ex situ ones JBB and OLO, are considerably inbred. One might speculate that both JBB and OLO would have “inherited” the inbreeding pattern characterizing the donor subpopulation over one decade ago, when seeds were collected. Although such a subpopulation does not exist at present (it was placed between the current ECM1 and ECM2 subpopulations), it would have suffered from the strong genetic substructuring of populations (i.e., Wahlund effect) that occurred in all populations at the beginning of this century: about 45% of the genetic diversity was due to differences between the subpopulations within each population (López-Pujol et al., 2002).

As noted in the “Results”, linkage disequilibrium is affecting all loci and most populations. This may indicate that this pattern, instead of being a consequence of physical linkages between chromosomes, is rather due to the occurrence of recent bottlenecks (genetic drift), in agreement with the results of the tests for recent bottlenecks provided in Table 5. Linkage disequilibrium is common in small populations (generally related with population bottlenecks), although population subdivision and consanguinity (as happens in cases of population fragmentation) may also lead to linkage disequilibrium (Zartman, McDaniel & Shaw, 2006; Slatkin, 2008).

### Levels of genetic diversity among populations and genetic structure

The *F*_ST_ values should be regarded as unexpectedly high in *S. farrenyi*, given that (1) populations are separated by just a few km, (2) they reproduce mainly by outcrossing, and (3) the gene flow could be favored by the strong northern winds prevailing in the region (*tramuntanades*) helping anemochory. This relative genetic isolation could, therefore, be interpreted as a sign of population fragmentation. It can be anticipated that these values could increase over the generations as the demographic bottlenecks are translated into genetic bottlenecks.

Finding two distinct genetic lineages in such a small geographic area (the most distant populations are separated by just three km) is a quite unexpected result, and we believe that it could be an early sign of population fragmentation in *S. farrenyi*. It is likely that all populations were connected in the past, either forming a macro-population which would include all extant populations and subpopulations, or with the presence of intervening populations or subpopulations, today extinct. The fragmentation between SES and ECM populations is fairly recent (in the 1990s there were still intermediate individuals between the two populations; J. Molero, 2002, personal communication). The BayesAss analysis, which has been used to explore the recent gene flow between the four genetic natural populations of *S. farrenyi* (SES2, ECM1, ECM2, and EBP) has revealed that there are still genetic exchanges between the eastern ones. The signs of such population fragmentation in *S. farrenyi* would have already been detectable in the former allozyme-based study (López-Pujol et al., 2002), as the presence of two lineages is observed if we run the software Structure with the raw allozyme data (Table S3). Similar to nSSR, the two largest populations of *S. farrenyi* (EBP and ECM) are roughly assigned to alternative genetic clusters, while SES1 shows a pattern of admixture (Fig. S1). Today, subpopulations SES1 and SES2 are assigned to one of the two lineages (Fig. 2); such “fixation” is not an unexpected result given that in the *locus classicus* there were about 500 individuals in late 1970s, and in 1999 (1 year before the sampling for the allozyme study), the size of SES1 was still of 90 individuals (Table 1). The ongoing fragmentation of *S. farrenyi* populations, coupled with the extinction of intervening populations/subpopulations, may account for the absence of isolation-by-distance pattern.

As for the restoration of *S. farrenyi* (particularly if the recovery plan is readily implemented), it is important to take into account the existence of these two genetic lineages, already present almost two decades ago. As stated in the “Introduction”, the individuals used in a reintroduction can be inappropriate if the donor population is highly genetically divergent, because this might involve breakdown of the co-adapted gene complexes; such processes would lead to outbreeding depression (Fenster & Dudash, 1994) and, finally, might increase the probability of extinction (Hamrick & Godt, 1996). The genetic differences observed between the populations of *S. farrenyi* are probably not enough to trigger the aforementioned negative effects (Frankham et al., 2011), but at least, in a first phase of urgent measures of genetic rescue, it would be most convenient not to mix the genetic stocks of the two lineages (EBP/ECM-SES) as a precaution. Thus, we recommend the plant material of the three known ex situ populations (JBB and OLO, under the Catalan government administration, and that of the Botanical Garden of Mulhouse, managed by the local government) to be only used for the population reinforcement of the ECM and/or SES population. The ex situ population of JBB is the most suitable source of plant material when reinforcing ECM and SES populations, since it harbors six exclusive alleles plus two shared alleles with the other ex situ populations (one with OLO and another one with OLO and SES1). These eight alleles would have been present in ECM only a decade ago and their loss could be partly attributed to the massive collection of seeds that was carried out in 2008 (about 2200 seeds were collected; Martinell, 2011); one might even speculate that such seed collection would have caused the extinction of the subpopulation where the seeds were collected (which was located between ECM1 and ECM2 subpopulations). Plants with strictly sexual reproduction depend exclusively on seed production and, if they are monocarpic such as *S. farrenyi*, then seed yield is even more important for ensuring population viability. Other causes of allele loss could be attributed to catastrophic events due to violent episodes of sea storms with strong winds (locally named *llevantades*) and reported in the last 10 years from the same coastal strip (as on *Silene sedoides* Poir.—C. Blanché, 2019, personal observations) or in close coastal areas (e.g., on *Limonium perplexum* L. Sáez & Rosselló in the Valencian Country—Laguna et al. (2016), or on *Dorycnium fulgurans* (Porta) Lassen in Mallorca—Fenu et al. (2019), among many others). Extensive and unknown consequences (Davis et al., 2012) of climatic change on ecological properties (germination, drought resistance, pollination success, etc.) may even have contributed to a fitness loss driving to a fall in demographic numbers, ultimately resulting in an allele loss.

Future reinforcements to EBP, in contrast, should be carried out with material obtained from this population and not from other sources. Since *S. farrenyi* is a monocarpic species, seed collection should be done with extreme care, ensuring that only a small percentage of the total production of seeds of an individual is taken; the episodic falls on the ratio of reproductive/vegetative individuals (ca. 3% for the years 2014 and 2017 from an average of ca. 16% for the period 2010–2019; S. Saura-Mas & G. Carrión, 2019, personal communication) stresses the need to be even more cautious. Collecting all seeds of a given individual would involve most certainly the loss of one of the species’ multi-locus genotypes (as all the individuals from natural populations have their own genotype; see above), which could represent the loss of particular alleles in the case in which they were only present in that individual. Apart from urgent rescue actions, creating an experimental (ex situ) population is also highly recommended, where stocks coming from the two identified genetic lineages would be mixed, and where individual fitness and demographic viability can be compared with those of non-admixture reinforced populations. According to Frankham et al. (2011), the risk of outbreeding depression would be limited when the populations have been isolated for <500 years, and that occupy a similar environment, which is the case of *S. farrenyi*.

## Conclusions

Our main finding is that levels of genetic diversity in the natural populations of *S. farrenyi* are still high, in spite of its narrow range and its accelerated population decline. Genetic data reported in this article have resulted (unintentionally) in a first contribution to a genetic monitoring program. Our results also demonstrated, again unintentionally, the value of ex situ collections in conservation genetics, as several alleles that are presumably lost in natural populations have been conserved, thanks to the germplasm and living collections of botanical gardens. This being the starting point, a recovery plan could include both wild subpopulations and the new experimentally created mixed population as monitoring subjects, because *S. farrenyi* has shown enough variation in genetic markers along time to reveal significant changes. An adequate managing of the system of wild/experimental populations can provide the recovery of genetic diversity levels. If genetic indicators are included in recovery success evaluation, the project could thus be considered as a genetic reserve within a protected area (Iriondo et al., 2008) for wild species.

## Supplemental Information

10.7717/peerj.10521/supp-1 Supplemental Information 1 *Seseli farreny*i population analysis using Structure. Raw allozyme data (Table S3) was generated in 2000 and used in López-Pujol et al. (2002).

10.7717/peerj.10521/supp-2 Supplemental Information 2 Allelic frequencies for the nine polymorphic loci analyzed within the populations of *Seseli farrenyi*.

10.7717/peerj.10521/supp-3 Supplemental Information 3 Mean recent migration rates (*m*) and 95% confidence interval among the natural populations of *Seseli farrenyi* estimated from nine nSSR data using the BayesAss program.

10.7717/peerj.10521/supp-4 Supplemental Information 4 Raw allozyme data of *Seseli farrenyi* generated in 2000, used in López-Pujol et al. (2002).

## Additional Information and Declarations

### Field Study Permissions

The following information was supplied relating to field study approvals (i.e., approving body and any reference numbers): Permissions to access to the populations and sample the plant material were provided by the Catalonian Government (“Generalitat de Catalunya”).

### DNA Deposition

The following information was supplied regarding the deposition of DNA sequences: Sequences are available in Table 3 and at GenBank: MT628686, MT628687, MT628688, MT628689, MT628690, MT628691, MT628692, MT628693, MT628694, MT628695, MT628696, MT628697, MT628698, MT628699.

### Data Availability

The following information was supplied regarding data availability: The allelic frequencies for the nine polymorphic loci are available in the Supplemental Files.
