# Supplementary material for: Genetic diversity and structure of the narrow endemic Seseli farrenyi (Apiaceae): implications for translocation
Source: PeerJ. 2021 Feb 4;9:e10521. doi: 10.7717/peerj.10521 (PMC7868069; doi:10.7717/peerj.10521)
Supplement: Supplemental Information 1 — Raw allozyme data (Table S3) was generated in 2000 and used in López-Pujol et al. (2002). [file peerj-09-10521-s001.pdf]

Figure S1. Analysis of *Seseli farrenyi* populations with Structure v. 2.3.4 (*Pritchard, Stephens & Donnelly, 2000*), using the raw allozyme data (Table S3) generated in the year 2000 and which were employed for producing the study of *López-Pujol et al. (2002)*. In this analysis, the dataset is much larger (*N* = 277) as in the year 2000 the total population size was estimated to be around 2000 (*López-Pujol, 2000*; see also Table 1). As with nSSR, fhe final image of this analysis was obtained using the programs Clumpp v. 1.1.2 (*Jakobsson & Rosenberg, 2007*) and Distruct v. 1.1 (*Rosenberg, 2004*), assuming *K* = 2 and *K* = 3.

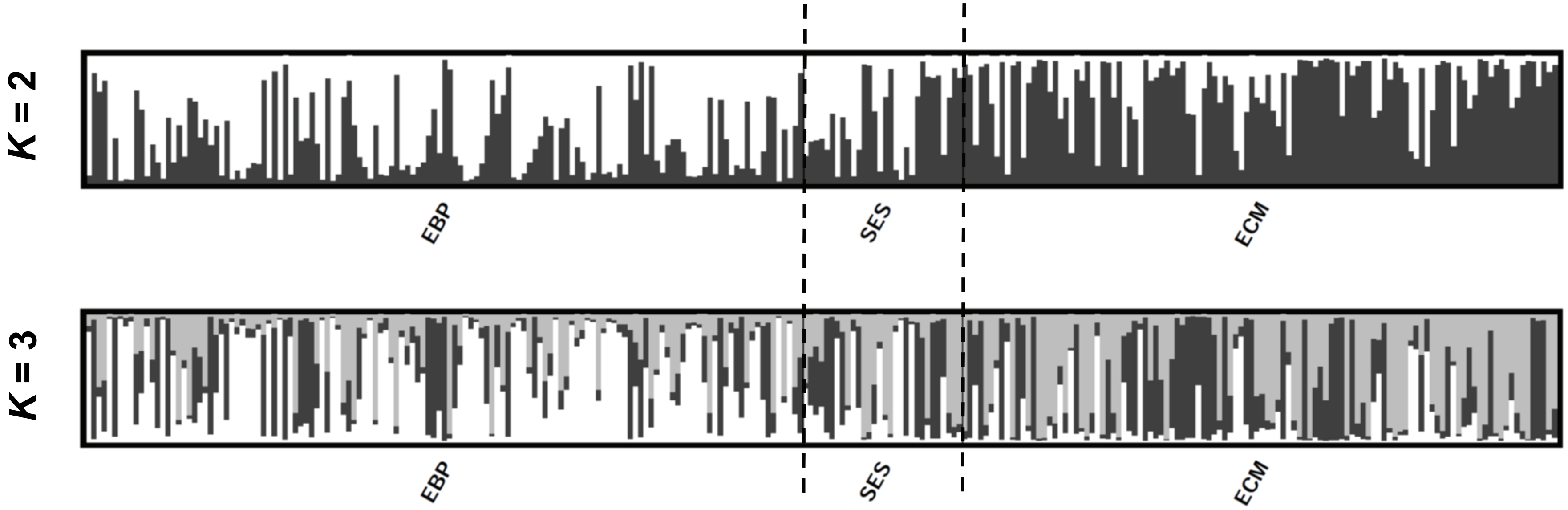

Jakobsson M, Rosenberg NA. 2007. CLUMPP: a cluster matching and permutation program for dealing with label switching and multimodality in analysis of population structure. *Bioinformatics* **23(14)**:1801–1806 DOI 10.1093/bioinformatics/btm233.

López-Pujol J. 2000. Diversitat isoenzimàtica en dues espècies endèmiques de Catalunya: *Petrocoptis montsicciana* i *Seseli farrenyi*. M. Sc. Dissertation, Universitat de Barcelona.

López-Pujol J, Bosch M, Simon J, Blanché C. 2002. Allozyme variation and population structure of the very narrow endemic *Seseli farrenyi* (Apiaceae). *Botanical Journal of the Linnean Society* **138(3)**:305–314 DOI 10.1046/j.1095-8339.2002.00021.x.

Pritchard JK, Stephens M, Donnelly P. 2000. Inference of population structure using multilocus genotype data. *Genetics* **155(2)**:945–959.

Rosenberg NA. 2004. Distruct: a program for the graphical display of population structure. *Molecular Ecology Notes* 4(1):137–138 DOI 10.1046/j.1471-8286.2003.00566.x.
