# Supplementary material for: Genetic diversity and structure of the narrow endemic Seseli farrenyi (Apiaceae): implications for translocation
Source: PeerJ. 2021 Feb 4;9:e10521. doi: 10.7717/peerj.10521 (PMC7868069; doi:10.7717/peerj.10521)
Supplement: Supplemental Information 2 [file peerj-09-10521-s002.docx]

**Table S1.** Allelic frequencies for the nine polymorphic loci analysed within the populations of *Seseli farrenyi*. In italics, rare alleles (frequency <0.05); in bold, private alleles (exclusive to a given population); gray-shaded, alleles only found in ex situ populations; underlined, the most frequent allele per locus and population.

| **Locus** | **Allele** | **Natural populations** | | | | | **Ex situ populations** | | |
| --- | --- | --- | --- | --- | --- | --- | --- | --- | --- |
|  |  | **SES2** | **ECM1** | **ECM2** | **ECM^1^** | **EBP** | **SES1** | **JBB** | **OLO** |
| **SFA2** | 154 | 0.000 | 0.267 | 0.333 | 0.286 | 0.132 | 0.000 | 0.180 | 0.000 |
|  | 156 | 0.167 | 0.467 | 0.500 | 0.476 | 0.421 | 1.000 | 0.720 | 0.875 |
|  | 172 | 0.000 | 0.000 | 0.083 | *0.024* | 0.132 | 0.000 | 0.000 | 0.000 |
|  | 174 | 0.000 | 0.067 | 0.083 | 0.071 | 0.237 | 0.000 | 0.100 | 0.125 |
|  | 176 | 0.000 | 0.000 | 0.000 | 0.000 | **0.079** | 0.000 | 0.000 | 0.000 |
|  | 178 | 0.833 | 0.200 | 0.000 | 0.143 | 0.000 | 0.000 | 0.000 | 0.000 |
| **SFA12** | 194 | 0.000 | 0.000 | 0.000 | 0.000 | **0.237** | 0.000 | 0.000 | 0.000 |
|  | 202 | 0.333 | 0.000 | 0.083 | *0.024* | 0.053 | 0.500 | 0.100 | 0.000 |
|  | 204 | 0.000 | 0.100 | 0.083 | 0.095 | 0.000 | 0.000 | 0.300 | 0.500 |
|  | 208 | 0.000 | 0.000 | **0.083** | ***0.024*** | 0.000 | 0.000 | 0.000 | 0.000 |
|  | 210 | 0.000 | 0.000 | 0.000 | 0.000 | 0.000 | 0.500 | 0.060 | 0.125 |
|  | 212 | 0.667 | 0.067 | 0.000 | *0.048* | 0.000 | 0.000 | 0.000 | 0.000 |
|  | 214 | 0.000 | 0.100 | 0.250 | 0.143 | 0.224 | 0.000 | *0.040* | 0.000 |
|  | 216 | 0.000 | 0.200 | 0.000 | 0.143 | *0.026* | 0.000 | 0.300 | 0.208 |
|  | 218 | 0.000 | 0.000 | 0.000 | 0.000 | **0.289** | 0.000 | 0.000 | 0.000 |
|  | 220 | 0.000 | 0.000 | 0.000 | 0.000 | ***0.013*** | 0.000 | 0.000 | 0.000 |
|  | 234 | 0.000 | 0.000 | 0.500 | 0.143 | 0.158 | 0.000 | *0.040* | 0.000 |
|  | 236 | 0.000 | 0.533 | 0.000 | 0.381 | 0.000 | 0.000 | 0.160 | 0.167 |
| **SFA15** | 179 | 0.333 | 0.200 | 0.417 | 0.262 | 0.092 | 0.500 | 0.300 | 0.333 |
|  | 180 | 0.000 | 0.000 | 0.000 | 0.000 | ***0.026*** | 0.000 | 0.000 | 0.000 |
|  | 181 | 0.000 | 0.300 | 0.000 | 0.214 | *0.026* | 0.000 | 0.240 | 0.250 |
|  | 183 | 0.000 | ***0.033*** | **0.083** | ***0.048*** | 0.000 | 0.000 | 0.000 | 0.000 |
|  | 185 | 0.000 | 0.000 | 0.000 | 0.000 | ***0.013*** | 0.000 | 0.000 | 0.000 |
|  | 187 | 0.500 | 0.067 | 0.000 | *0.048* | 0.000 | 0.500 | 0.120 | 0.167 |
|  | 189 | 0.000 | 0.133 | 0.083 | 0.119 | 0.289 | 0.000 | *0.020* | 0.083 |
|  | 191 | 0.000 | 0.133 | 0.250 | 0.167 | *0.013* | 0.000 | 0.140 | *0.042* |
|  | 193 | 0.167 | 0.000 | 0.000 | 0.000 | 0.053 | 0.000 | 0.000 | 0.000 |
|  | 195 | 0.000 | 0.000 | 0.083 | *0.024* | 0.092 | 0.000 | 0.000 | 0.000 |
|  | 197 | 0.000 | 0.133 | 0.000 | 0.095 | 0.053 | 0.000 | 0.180 | 0.125 |
|  | 199 | 0.000 | 0.000 | 0.083 | *0.024* | 0.224 | 0.000 | 0.000 | 0.000 |
|  | 209 | 0.000 | 0.000 | 0.000 | 0.000 | **0.118** | 0.000 | 0.000 | 0.000 |
| **SFA18** | 149 | 0.000 | 0.000 | 0.000 | 0.000 | **0.092** | 0.000 | 0.000 | 0.000 |
|  | 151 | 0.000 | 0.000 | 0.000 | 0.000 | 0.158 | 0.000 | *0.020* | 0.000 |
|  | 153 | 0.000 | 0.000 | 0.000 | 0.000 | **0.053** | 0.000 | 0.000 | 0.000 |
|  | 155 | 0.667 | 0.200 | 0.417 | 0.262 | 0.053 | 0.000 | 0.580 | 0.667 |
|  | 157 | 0.000 | 0.567 | 0.500 | 0.548 | *0.013* | 0.000 | 0.220 | 0.167 |
|  | 159 | 0.000 | 0.233 | 0.083 | 0.190 | 0.118 | 0.000 | 0.180 | 0.167 |
|  | 161 | 0.167 | 0.000 | 0.000 | 0.000 | 0.224 | 0.000 | 0.000 | 0.000 |
|  | 163 | 0.167 | 0.000 | 0.000 | 0.000 | 0.092 | 1.000 | 0.000 | 0.000 |
|  | 167 | 0.000 | 0.000 | 0.000 | 0.000 | ***0.026*** | 0.000 | 0.000 | 0.000 |
|  | 169 | 0.000 | 0.000 | 0.000 | 0.000 | **0.171** | 0.000 | 0.000 | 0.000 |
| **SFA24** | 246 | 0.000 | 0.000 | 0.000 | 0.000 | **0.211** | 0.000 | 0.000 | 0.000 |
|  | 248 | 0.000 | 0.000 | 0.000 | 0.000 | ***0.039*** | 0.000 | 0.000 | 0.000 |
|  | 250 | 0.333 | *0.033* | 0.500 | 0.167 | 0.250 | 1.000 | 0.100 | 0.167 |
|  | 254 | 0.000 | 0.000 | 0.000 | 0.000 | **0.053** | 0.000 | 0.000 | 0.000 |
|  | 260 | 0.000 | 0.000 | 0.000 | 0.000 | ***0.013*** | 0.000 | 0.000 | 0.000 |
|  | 262 | 0.000 | 0.267 | 0.167 | 0.238 | 0.105 | 0.000 | 0.200 | 0.417 |
|  | 264 | 0.667 | 0.700 | 0.333 | 0.595 | 0.303 | 0.000 | 0.700 | 0.417 |
|  | 274 | 0.000 | 0.000 | 0.000 | 0.000 | ***0.026*** | 0.000 | 0.000 | 0.000 |
| **SFA31** | 216 | 0.833 | 1.000 | 1.000 | 1.000 | 0.303 | 0.500 | 0.980 | 1.000 |
|  | 218 | 0.000 | 0.000 | 0.000 | 0.000 | **0.289** | 0.000 | 0.000 | 0.000 |
|  | 220 | 0.167 | 0.000 | 0.000 | 0.000 | 0.000 | 0.500 | 0.000 | 0.000 |
|  | 222 | 0.000 | 0.000 | 0.000 | 0.000 | **0.184** | 0.000 | 0.000 | 0.000 |
|  | 224 | 0.000 | 0.000 | 0.000 | 0.000 | 0.224 | 0.000 | *0.020* | 0.000 |
| **SFA32** | 181 | 0.000 | 0.000 | 0.000 | 0.000 | ***0.014*** | 0.000 | 0.000 | 0.000 |
|  | 185 | 0.000 | 0.321 | 0.500 | 0.375 | 0.056 | 0.000 | 0.174 | 0.000 |
|  | 187 | 0.000 | 0.000 | 0.000 | 0.000 | ***0.028*** | 0.000 | 0.000 | 0.000 |
|  | 193 | 0.000 | 0.000 | 0.000 | 0.000 | 0.000 | 0.000 | ***0.043*** | 0.000 |
|  | 195 | 0.000 | 0.000 | 0.000 | 0.000 | 0.000 | 0.000 | *0.043* | 0.091 |
|  | 197 | 0.000 | *0.036* | 0.167 | 0.075 | 0.069 | 0.000 | 0.087 | 0.318 |
|  | 199 | 0.000 | 0.000 | 0.000 | 0.000 | *0.042* | 0.000 | *0.043* | 0.182 |
|  | 201 | 0.000 | 0.179 | 0.167 | 0.175 | 0.375 | 0.000 | 0.217 | 0.227 |
|  | 203 | 0.000 | 0.107 | 0.000 | 0.075 | 0.056 | 0.000 | *0.043* | 0.000 |
|  | 205 | 0.000 | 0.000 | 0.000 | 0.000 | ***0.014*** | 0.000 | 0.000 | 0.000 |
|  | 207 | 0.000 | *0.036* | 0.000 | *0.025* | 0.181 | 0.000 | *0.043* | 0.000 |
|  | 209 | 1.000 | 0.214 | 0.167 | 0.200 | *0.028* | 0.000 | 0.261 | 0.091 |
|  | 211 | 0.000 | 0.071 | 0.000 | 0.050 | 0.056 | 0.000 | *0.043* | 0.091 |
|  | 213 | 0.000 | 0.000 | 0.000 | 0.000 | ***0.028*** | 0.000 | 0.000 | 0.000 |
|  | 219 | 0.000 | *0.036* | 0.000 | *0.025* | 0.056 | 1.000 | 0.000 | 0.000 |
| **SFA35** | 182 | 0.000 | 0.000 | 0.000 | 0.000 | 0.000 | 0.000 | ***0.040*** | 0.000 |
|  | 186 | 0.333 | 0.367 | 0.333 | 0.357 | 0.132 | 0.500 | 0.260 | 0.333 |
|  | 188 | 0.000 | 0.167 | 0.083 | 0.143 | 0.000 | 0.000 | 0.280 | 0.167 |
|  | 192 | 0.000 | 0.000 | 0.000 | 0.000 | ***0.013*** | 0.000 | 0.000 | 0.000 |
|  | 194 | 0.500 | 0.100 | 0.000 | 0.071 | 0.000 | 0.500 | 0.160 | 0.167 |
|  | 196 | 0.000 | 0.167 | 0.083 | 0.143 | 0.342 | 0.000 | 0.060 | 0.083 |
|  | 198 | 0.167 | 0.067 | 0.250 | 0.119 | *0.026* | 0.000 | *0.040* | *0.042* |
|  | 200 | 0.000 | 0.000 | 0.000 | 0.000 | **0.145** | 0.000 | 0.000 | 0.000 |
|  | 202 | 0.000 | 0.133 | 0.167 | 0.143 | 0.000 | 0.000 | 0.160 | 0.208 |
|  | 204 | 0.000 | 0.000 | 0.083 | *0.024* | 0.342 | 0.000 | 0.000 | 0.000 |
| **SFA48** | 153 | 0.000 | 0.000 | 0.000 | 0.000 | 0.000 | 0.000 | ***0.040*** | 0.000 |
|  | 155 | 0.000 | 0.000 | 0.000 | 0.000 | 0.000 | 0.000 | ***0.040*** | 0.000 |
|  | 157 | 0.000 | 0.000 | 0.000 | 0.000 | 0.000 | 0.000 | ***0.020*** | 0.000 |
|  | 159 | 0.000 | **0.100** | 0.000 | **0.071** | 0.000 | 0.000 | *0.040* | 0.000 |
|  | 161 | 1.000 | 0.000 | 0.000 | 0.000 | *0.039* | 0.000 | 0.080 | 0.000 |
|  | 163 | 0.000 | 0.067 | 0.500 | 0.190 | 0.000 | 0.000 | 0.160 | 0.417 |
|  | 165 | 0.000 | 0.667 | 0.333 | 0.571 | 0.132 | 1.000 | 0.300 | 0.417 |
|  | 167 | 0.000 | 0.000 | 0.167 | *0.048* | 0.105 | 0.000 | *0.040* | 0.083 |
|  | 169 | 0.000 | 0.000 | 0.000 | 0.000 | 0.447 | 0.000 | 0.000 | 0.083 |
|  | 171 | 0.000 | 0.000 | 0.000 | 0.000 | ***0.026*** | 0.000 | 0.000 | 0.000 |
|  | 173 | 0.000 | 0.167 | 0.000 | 0.119 | 0.092 | 0.000 | 0.260 | 0.000 |
|  | 179 | 0.000 | 0.000 | 0.000 | 0.000 | 0.000 | 0.000 | ***0.020*** | 0.000 |
|  | 181 | 0.000 | 0.000 | 0.000 | 0.000 | **0.158** | 0.000 | 0.000 | 0.000 |

^1^ ECM is the combination of ECM1 and ECM2
