# Supplementary material for: Genetic diversity and structure of the narrow endemic Seseli farrenyi (Apiaceae): implications for translocation
Source: PeerJ. 2021 Feb 4;9:e10521. doi: 10.7717/peerj.10521 (PMC7868069; doi:10.7717/peerj.10521)
Supplement: Supplemental Information 3 [file peerj-09-10521-s003.docx]

**Table S2.** Mean recent migration rates (*m*) and 95% confidence interval among the natural populations of *Seseli farrenyi* estimated from nine nSSR data using the BayesAss program. Values on the diagonal (underlined) are the proportions of individuals in each generation that are not migrants. Simulations in BayesAss show that in instances where there is no information in the data, the mean *m* and 95% confidence interval for datasets of four populations are 0.0553 and 0.0002–0.2180, respectively; values in bold are the *m* rates that are informative.

|  | From | | | |
| --- | --- | --- | --- | --- |
| To | SES2 | ECM1 | ECM2 | EBP |
| SES2 | 0.786 (0.670–0.991) | **0.142** (0.001–0.292) | 0.036 (0.000–0.144) | 0.037 (0.000–0.148) |
| ECM1 | 0.007 (0.000–0.038) | 0.978 (0.923–0.999) | 0.008 (0.000–0.043) | 0.007 (0.000–0.033) |
| ECM2 | 0.027 (0.000–0.109) | **0.232** (0.113–0.313) | 0.714 (0.668–0.824) | 0.027 (0.000–0.110) |
| EBP | 0.005 (0.000–0.024) | 0.003 (0.000–0.018) | 0.006 (0.000–0.024) | 0.986 (0.957–0.999) |
